# Relationship between readiness for interprofessional learning and academic self-efficacy among nursing students: a cross-sectional study

**DOI:** 10.1186/s12909-023-04953-3

**Published:** 2024-01-15

**Authors:** Ran An, Jinfang Wang, Shaojie Li, Na Li, Yongtian Yin, Xinyuan Wang

**Affiliations:** 1 School of Nursing, Shandong University of Traditional Chinese Medicine, Wendong Street, Jinan, Lixia District China; 2 School of Public Health, Peking University Health Science Center, Xueyuan Road, Beijing, Haidian District China; 3 Department of Education, Shandong Normal University, Wendong Street, Jinan, Lixia District China

**Keywords:** Interprofessional learning readiness, Academic self-efficacy, Nursing students, Interprofessional education

## Abstract

**Background:**

Modern medicine emphasizes that medical professionals engage in interprofessional collaboration to better meet the diverse needs of patients from physical, psychological, and social perspectives. As nursing students are the future reserve of the clinical nursing workforce, nursing educators worldwide should pay close attention to nursing students’ interprofessional learning attitudes and take responsibility for training qualified interprofessional nursing personnel. However, little is known about the relationship between nursing students' readiness for interprofessional learning and academic self-efficacy. Thus, this study aims to investigate the level of readiness for interprofessional learning and academic self-efficacy among nursing students, and to explore the relationship between the two.

**Methods:**

A cross-sectional survey was conducted with a sample of 741 undergraduate nursing students pursuing four-year degrees from a school in Jinan, Shandong Province, China from November to December 2021. The social-demographic questionnaire, Readiness for Interprofessional Learning Scale, and Academic Self-efficacy Scale were used for data collection. Descriptive statistics used to analyze the data included: Cronbach's alpha, t-test, one-way ANOVA, Pearson’s correlation, and multiple linear regression analysis.

**Results:**

Readiness for interprofessional learning mean score was (3.91 ± 0.44) and mean academic self-efficacy was (3.47 ± 0.42). Significant differences were found in the research variables according to participants’ sex, grade, choice of nursing profession, and frequency of communication with health-related major students in studies (*p* < 0.05, *p* < 0.001). Pearson correlation analysis showed that academic self-efficacy was positively related to readiness for interprofessional learning (*r* = 0.316, *p* < 0.01). The hierarchical regression analysis showed that academic self-efficacy was positively related to readiness for interprofessional learning (β = 0.307, *p* < 0.001), The model explained 15.6% of the variance in readiness for interprofessional learning (F = 18.038, *p* < 0.001).

**Conclusions:**

Readiness for interprofessional learning and academic self-efficacy were in the middle level among nursing students. Moreover, there was a significant positive correlation between the two. Therefore, it is very important for nursing educators to improve nursing students’ academic self-efficacy before improving their readiness for interprofessional learning.

## Background

With the continuous development of medical and health technologies and rapid transformation in society’s demographic structure and disease spectrum, the medical environment has become increasingly complex and public health demands change frequently. In 2010, the World Health Organization proposed that medicine should strengthen interprofessional education for nursing students in the new century to better cope with complex health problems and meet public health needs [1].

Readiness for interprofessional learning is an attitude in assessing an individual to receive interprofessional education. It refers to an individual’s willingness to cooperate with people from different professional backgrounds, participate in task decisions, and achieve learning goals [2]. Nursing students actively participate in interprofessional teamwork, enhancing their interaction and communication with medical, pharmacy, and other healthcare students. This teamwork helps define their professional roles more clearly, effectively reducing the risk of errors caused by clinical communication failures. Furthermore, it provides patients with better nursing service quality [3]. Nursing students are the foundation for expanding clinical nursing teams, thus, they should develop their interprofessional learning skills and attitude to lay the foundation for future interprofessional teamwork.

Several studies have reported that nursing students have different readiness levels for interprofessional learning across regions due to differences in socio-cultural background or learning environment..For example, the readiness for interprofessional learning scores of 260 Iranian nursing students and 738 Turkish nursing students were 82.40 ± 23.16 and 69.78 ± 11.32, respectively [4, 5]. By contrast, the readiness for interprofessional learning score of 928 Chinese nursing students lay in the middle, at 74.09 ± 10.54 [6]. However, the study settings were institutions practicing Western medicine, and there is no information on nursing students’ readiness for interprofessional learning at traditional Chinese medicine universities (TCMUs). Therefore, it is important to explore this aspect.

Researchers have explored many factors affecting nursing students’ readiness for interprofessional learning; however, the findings have not been consistent. For example, Zhang et al. [6]. Found that female students are more ready for interprofessional learning than male students; however, another study found that sex was not associated with readiness for interprofessional learning [7]. External factors such as grade, educational background, and clinical learning environment, as well as internal factors such as psychological capital, role stress, and personality traits, affect nursing students’ readiness for interprofessional learning [8–10]. However, existing research has rarely explored the relationship between academic self-efficacy and nursing students' readiness for interprofessional learning [11].

Academic self-efficacy is an individual’s perception of controlling their learning ability and behavior, reflecting their internal beliefs [12]. Bandura [13] stated that academic self-efficacy is not innate but depends on the gradual cultivation and accumulation of various learning environments or activities. It is an intrinsic motivation that enhances students’ self-directed learning. However, the teaching method in Chinese schools has changed in accordance with the changes in the pandemic’s severity. The combination of traditional face-to-face and online classes has led to a loss in stability of learning form and environment, seriously affecting students’ learning enthusiasm [14]. Wang et al. [14] found that 39.29% of Chinese nursing students had some risk of academic burnout during the COVID-19 pandemic. However, their level of academic self-efficacy remains unknown.

A US study revealed that nursing students involved in interprofessional collaborative simulations showed increased confidence and self-efficacy when caring for patients [11]. Meanwhile, several studies have shown a significant correlation between interprofessional learning and self-efficacy, including informational, occupational, and collaborative self-efficacy [11, 15, 16]. The studies indicated that nursing students' academic self-efficacy was positively correlated with their readiness for self-directed learning, and that academic self-efficacy may also benefit their attitudes toward learning [17, 18]. Nursing students with higher academic self-efficacy tend to be more prepared for learning and have more positive attitudes toward learning. Another study reported that students' self-reflection and belief play a crucial role in readiness for interprofessional learning [19]. Students with high levels of self-reflection and beliefs may be more focused on learning goals or have a higher motivation to invest in readiness for interprofessional learning. Through the above, we find that more studies have explored the correlation of one variable in academic self-efficacy or readiness for interprofessional learning with other variables. There are few reports on the relationship between readiness for interprofessional learning and academic self-efficacy.

Social cognitive theory can explain the social learning process, mainly emphasizing the dominant role of beliefs, expectations, and motivation on individual behavior [20]. It is believed that personal and environmental factors are important influences on individual behavior. Academic self-efficacy, as a personal belief factor, may interact with nursing students' learning environment to influence their interprofessional learning behaviors. In addition, owing to the pandemic, dynamic changes in teaching methods may change nursing students' enthusiasm for interprofessional learning by affecting their learning autonomy. Therefore, it is necessary to explore the inherent relationship between readiness for interprofessional learning and academic self-efficacy to make nursing educators intervene early.

Based on the above, this study has the following aims:

1. To explore the level of readiness for interprofessional learning and academic self-efficacy in Chinese nursing students.
2. To examine the relationship between readiness for interprofessional learning and academic self-efficacy in nursing students.

## Methods

### Design

A cross-sectional, descriptive study was conducted from November to December 2021 to clarify the relationship between readiness for interprofessional learning and academic self-efficacy in Chinese nursing students.

### Participants and procedures

This study used a convenience sampling method to select freshmen to senior year nursing students from a school in Jinan City, Shandong Province, China. Paper and online questionnaires were used to collect data. We began by obtaining informed consent from the student work department of the school and then informed the students of the purpose and significance of this study. After obtaining both verbal and written consent, we distributed questionnaires to those students who agreed to participate. Inclusion criteria were as follows: full-time undergraduate nursing student, age ≥ 18 years, and provided informed consent to participate in the study voluntarily. Exclusion criteria were as follows: students with severe mental illness, such as major depression, and those unable to complete the questionnaire for various reasons. A total of 223 nursing students were excluded from this study.

The researchers and two trained research assistants entered the classroom to administer questionnaires on-site. However, the senior nursing students were practicing in various hospitals during the survey period; therefore, we used the online platform Questionnaire Star to administer surveys to those students. In China, Questionnaire Star is a professional online questionnaire survey platform with the obvious advantages of being fast, easy to use, and inexpensive. During this survey, we received 800 questionnaires (freshmen: 238; sophomore: 256; junior: 269; senior: 37). After excluding the vacant items and the invalid items of the same scale (the answer results were the same for 10 consecutive times or regular answers), 741 questionnaires were valid, with an effective response rate of 92.63%.

### Instruments

#### Social-demographic information questionnaire

The socio-demographic information questionnaire included seven items on participants’ sex, age, grade, only-child status (only children will be more introverted compared to those with siblings, due to lack of communication with peers in the family [21]), choice of nursing profession, situation of learning stress, and frequency of communication with students majoring in other health-related subjects.

#### Readiness of Interprofessional Learning Scale (RIPLS)

The RIPLS was published by British scholars Parsell and Bligh [22] and translated for Chinese use by Wang and Hu [23]. The scale includes four dimensions: teamwork and collaboration (9 items), negative professional identity (3 items), positive professional identity (4 items), and roles and responsibilities (3 items), with a total of 19 items. Responses were assessed on a 5-point Likert scale ranging from 1 (*strongly disagree*) to 5 (*strongly agree*). Total RIPLS scores range from 19 ~ 95, with higher scores indicating greater individual readiness for interprofessional learning. In this study, Cronbach’s alpha of the RIPLS was 0.894.

#### Academic Self-Efficacy Scale (ASES)

The ASES was developed by Liang [24] and consists of two dimensions—self-efficacy for learning ability (11 items) and self-efficacy for learning behavior (11 items)—with 22 items in total. Responses were rated on a 5-point Likert scale ranging from 1 (*totally inconsistent*) to 5 (*totally consistent*). Total ASES scores range from 22 ~ 110, with higher scores indicating greater academic self-efficacy. In this study, Cronbach’s alpha of the ASES was 0.862.

### Data analysis

Data analysis was performed using SPSS 25.0. Normally distributed measurement data were expressed as mean ± SD, and count data were expressed as frequency and percentage. Differences in readiness for interprofessional learning and academic self-efficacy scores were analyzed using t-test and one-way ANOVA. Further, the relationship between the two variables was analyzed using Pearson’s correlation. Hierarchical regression analysis was used to further analyze the relationship between academic self-efficacy and readiness for interprofessional learning. In regression model 1, the potential confounding variables in this study were included. More specifically, sex, age, grade, only-child status, choice of nursing profession, situation of learning stress, and frequency of communication with health-related major students were placed into the first level of the regression model. In regression model 2, academic self-efficacy was added. The dependent variable was readiness for interprofessional learning. The level of significance was set at 0.05 for all two-tailed t-tests.

## Results

### Descriptive statistics of social-demographic information

Participants’ demographic data are presented in Table 1. A total of 741 nursing students participated in the study, with an average age of 19.60 years (SD = 1.13). Overall, 82.46% (*n* = 611) participants were female, approximately 34.82% (*n* = 258) were juniors, and 25.91% (*n* = 192) were the only child in their families. Further, 62.75% (*n* = 465) actively chose the nursing profession, 27.53% (*n* = 204) experienced high learning pressure, and 76.65% (*n* = 568) occasionally communicated with students with other health-related majors.

**Table 1:** Participants’ social-demographic information (*n* = 741)

Variable	N (%)	Mean	SD	Range
Sex				
Female	611 (82.46%)			
Male	130 (17.54%)			
Age		19.60	1.13	18–24
Grade				
Freshman	222 (29.96%)			
Sophomore	233 (31.44%)			
Junior	258 (34.82%)			
Senior	28 (3.78%)			
Only-child status				
Yes	192 (25.91%)			
No	549 (74.09%)			
Choice of nursing profession				
Chose actively	465 (62.75%)			
Transfer	276 (37.25%)			
Low	69 (9.31%)			
Moderate	468 (63.16%)			
High	204 (27.53%)			
Frequency of communication with health-related major students				
Never	111 (14.98%)			
Occasionally	568 (76.65%)			
Regularly	62 (8.37%)			

### Descriptive statistics of readiness for interprofessional learning and academic self-efficacy

Table 2 presents the participants’ descriptive statistics of readiness for interprofessional learning and academic self-efficacy. The mean readiness score for interprofessional learning was 3.91 ± 0.44, with positive professional identity scoring the highest at 4.14 ± 0.60. The mean score of academic self-efficacy was 3.47 ± 0.42, with learning ability self-efficacy scoring the highest at 3.61 ± 0.56.

**Table 2:** Means, standard deviations, minimum, and maximum of readiness for interprofessional learning and academic self-efficacy (*n* = 741)

Variable	Score	Items	Mean score
Readiness for Interprofessional Learning	74.33 ± 8.35	19	3.91 ± 0.44
Teamwork and Collaboration	37.11 ± 5.05	9	4.12 ± 0.56
Negative Professional Identity	12.01 ± 2.30	3	4.00 ± 0.77
Positive Professional Identity	16.56 ± 2.40	4	4.14 ± 0.60
Roles and Responsibilities	8.66 ± 1.84	3	2.89 ± 0.61
Academic Self-efficacy	76.41 ± 9.29	22	3.47 ± 0.42
Learning Ability Self-efficacy	39.76 ± 6.11	11	3.61 ± 0.56
Learning Behavior Self-efficacy	36.65 ± 4.56	11	3.33 ± 0.41

### Distribution of scale mean scores according to characteristics of the nursing students

Table 3 presents the statistically significant differences in readiness for interprofessional learning among participants of different sexes, with female participants having higher RIPLS; grade, with RIPLS scores decreasing from freshman to senior level students; choice of nursing profession, with those actively choosing the profession scoring higher on the instrument; and frequency of communication with students with health-related majors, with those regularly communicating with other health professions students showing higher RIPLS scores (*p* < 0.05, *p* < 0.001).

**Table 3:** Distribution of scale mean scores according to characteristics of nursing students (*n* = 741)

Characteristics	RIPLS	ASES
	Mean ± SD	Mean ± SD
Sex		
Female	74.83 ± 8.05	76.56 ± 9.15
Male	71.98 ± 9.31	75.70 ± 9.91
*t*	3.565	0.964
*p*	< 0.001	0.335
Grade		
Freshman	76.60 ± 8.64	76.09 ± 9.31
Sophomore	74.07 ± 8.02	76.70 ± 10.32
Junior	72.91 ± 7.75	75.85 ± 7.59^b^
Senior	71.50 ± 10.20^b^	81.86 ± 12.47
*F*	9.429	3.736
*p*	< 0.001	0.011
Only-child status		
Yes	74.59 ± 8.67	76.33 ± 10.42
No	74.24 ± 8.24	76.44 ± 8.87
*t*	0.510	0.138
*p*	0.610	0.890
Choice of nursing profession		
Chose actively	75.08 ± 8.39	76.99 ± 9.33
Transfer	73.07 ± 8.15	75.44 ± 9.15
*t*	3.176	2.207
*p*	0.002	0.028
Situation of learning stress		
Low	75.38 ± 10.21	78.23 ± 10.49
Medium	74.25 ± 8.19	76.30 ± 8.85
High	74.17 ± 8.03	76.06 ± 9.80
*F*	0.605	1.509
*P*	0.547	0.222
Frequency of communication with health-related major students		
Never	73.16 ± 7.94^b^	73.86 ± 8.89^b^
Occasionally	74.24 ± 8.32	76.70 ± 9.39
Regularly	77.23 ± 8.79	78.39 ± 8.17
*F*	4.898	5.920
*P*	0.008	0.003

^b^Post hoc tests showed the score of this group was lower than the scores of the other groups

Among nursing students from different grade levels, ASES scores showed an increase over the course of the program. Furthermore, students who actively chose the profession also showed higher ASES scores. Finally, students who communicated more frequently with other health professions showed higher ASES scores. All three of these variables demonstrated statistically significant differences (*p* < 0.05).

### Correlations between readiness for interprofessional learning and academic self-efficacy

Pearson correlation analysis (Table 4) showed that academic self-efficacy was positively related to readiness for interprofessional learning (*r* = 0.316, *p* < 0.01). The hierarchical regression analysis results (Table 5) showed that academic self-efficacy was positively related to readiness for interprofessional learning (*β* = 0.307,* p* < 0.001) when controlling for sex, grade, choice of the nursing profession, and frequency of communication with students with other health majors. The model explained 15.6% of the variance in readiness for interprofessional learning (*F* = 18.038, *p* < 0.001).

**Table 4:** Correlation between readiness for interprofessional learning and academic self-efficacy in nursing students (*n* = 741)

	Learning Ability Self-efficacy	Learning Behavior Self-efficacy	Total ASES
Teamwork and Collaboration	0.335^b^	0.218^b^	0.327^b^
Negative Professional Identity	0.227^b^	-0.045	0.127^a^
Positive Professional Identity	0.391^b^	0.266^b^	0.388^b^
Roles and Responsibilities	-0.029	-0.228^b^	-0.131^b^
Total RIPLS	0.371^b^	0.146^b^	0.316^b^

^a^Correlation is significant at the 0.05 level (2-tailed)

^b^Correlation is significant at the 0.01 level (2-tailed)

**Table 5:** Hierarchical regression analysis of readiness for interprofessional learning (*n* = 741)

Variables	Step 1			Step 2		
	*β*	*t*	*p*	*β*	*t*	*p*
Constant		9.326	< 0.001***		6.928	< 0.001***
Sex	0.124	3.397	0.001**	0.118	3.387	0.001**
Age	-0.014	-0.242	0.809	-0.018	-0.325	0.745
Grade	-0.178	-3.033	0.003**	-0.187	-3.358	0.001**
Only-child status	-0.043	-1.186	0.236	-0.043	-1.246	0.213
Choice of nursing profession	-0.081	-2.257	0.024*	-0.060	-1.740	0.082
Situation of learning stress	-0.003	-0.089	0.929	0.012	0.344	0.731
Frequency of communication with health major students	0.102	2.830	0.005**	0.068	1.972	0.049*
Total ASES				0.307	8.966	< 0.001***
*R*^2^	0.064			0.156		
*F*	8.239***			18.038***		

*β* Standardized coefficients

^*^p < 0.05

^**^p < 0.01

^***^p < 0.001

## Discussion

This cross-sectional survey study explored the readiness levels for interprofessional learning and academic self-efficacy in Chinese nursing students and analyzed the relationship. Our findings can help nursing educators improve their strategies for teaching nursing, thus improving readiness for interprofessional learning and academic self-efficacy of students.

The findings showed that participants’ mean readiness score for interprofessional learning was 3.91 ± 0.44, which was at the upper average level compared with the mid-score of 3 points. These results were similar to those of medical and dental students in Nepal [25] (mean score 3.95), but higher than those of nursing students from non-TCMUs [5] (mean score 3.67). This could be because nursing students of TCMUs learn both Western and traditional Chinese medicine nursing techniques. As they are exposed to more diverse medical education, such as cupping, scraping, ear point pressure bean, and other nursing skills, they may be more inclined toward interprofessional learning.

Our study’s highest scoring dimension of readiness for interprofessional learning was positive professional identity, which differs from previous studies. For example, when Williams and Webb [26] investigated readiness for interprofessional learning among nursing students in nine Australian universities, they found that the highest scoring dimension was teamwork and collaboration. This may be related to the varied duration of the survey or the heterogeneity of the study population. Since the COVID-19 outbreak, central and regional governments have introduced several policies that favor healthcare workers. These include optimizing preferential treatment for healthcare workers and reporting on typical and touching stories from the front line. These effects triggered nursing students’ pride and recognition of the nursing profession. Additionally, 62.75% of the study participants volunteered to apply for nursing majors. Moreover, sufficient social and family support increase students’ identity in the nursing profession.

The study results showed that female participants had higher readiness for interprofessional learning than male participants, similar to the previous findings on American and Korean nursing students [27, 28]. This may be related to female personality characteristics and the professional nursing environment. One study has pointed out that women have more developed left-hemisphere function and are better at language expression and logical thinking compared to men [23]. Moreover, women have higher extroverted personality traits than men, and are often considered talkative, optimistic, and preferring to socialize in large groups [9]. It is suggested that universities should encourage male nursing students to actively participate in interprofessional learning and leverage the advantages of teamwork.

Higher-grade nursing students had lower readiness for interprofessional learning. This result is consistent with the survey of female health students conducted by Al-Eisa et al. [29] who found that as nursing students move through higher grades, their academic burden increases and their exposure to new things decreases. Over time, they may develop an “internal closure” mentality, which affects their willingness to participate in interprofessional learning [30].

Students who voluntarily chose the nursing profession had higher readiness for interprofessional learning. Research shows that nursing students who voluntarily choose the profession have a higher sense of professional identity [11]. They have strong professional learning motivation and are eager to acquire more knowledge and skills from other related majors through communication and cooperation, aiming to enrich their professional advantages and enhance their professional value. Additionally, our study found that nursing students who frequently communicate with students from other health-related majors had higher readiness for interprofessional learning, consistent with the findings of Lie et al. [31]. Therefore, in the future, university teachers should focus on the frequency of interaction between nursing students and students of other health-related majors, and build a interprofessional communication platform to increase the low-level interprofessional learning ability of nursing students.

This study showed that the average score of academic self-efficacy among nursing students was 3.47 ± 0.42, which was above average compared with the middle score of 3 among the items. However, it was lower than the academic self-efficacy of nursing students before the COVID-19 outbreak in China(3.53 ± 0.45) [32]. Previous studies also measured high levels of academic self-efficacy among nursing students in both the United States and Israel during the epidemic period [33, 34]. Nevertheless, it is difficult to compare other national studies’ results with the results of this study because of the different measurement tools used. Chinese scholars compiled the survey questionnaire used according to the learning characteristics of college students. It is a localized questionnaire suitable for investigating the academic self-efficacy of Chinese college students, with an internal consistency coefficient of 0.820 and high credibility [24]. In this survey, we found that nursing students had the lowest learning behavior self-efficacy scores. There are two possible reasons for this finding: the difference in the learning environment between universities and high schools and students’ learning styles and environments changed during the pandemic.

This study showed that senior nursing students had higher academic self-efficacy scores than freshmen, sophomores, and juniors, contrary to the findings on Korean nursing students by Kim et al. [35]. This may be related to the nursing employment environment in China. Seniors are close to graduation and face fierce recruitment competition; thus, they need to be more active in learning and have more nursing-related knowledge to stand out in the future employment environment. Moreover, before senior nursing students enter clinical practice, the school also takes a series of measures including pre-job training and skills assessment to help the students adapt to clinical practice, which may also promote students’ academic self-efficacy.

The higher academic self-efficacy of students who actively chose the nursing profession may be related to the fact that these students are more interested in the nursing profession and have higher autonomy in obtaining professional knowledge, thus generating higher academic self-efficacy [36]. However, students who do not actively choose the profession may be resistant to a nursing major and will show negative attitudes toward professional learning, resulting in lower academic self-efficacy.

Nursing students who often communicate with students of other health-related majors have higher academic self-efficacy. This may be because students from different majors exchange knowledge and have professional characteristics that are mutually attractive. The peer effect improves nursing students’ learning compliance and enhances their academic self-efficacy [37].

This study showed that nursing students’ readiness for interprofessional learning was positively associated with academic self-efficacy (*p* < 0.01), indicating that higher academic self-efficacy predicted a more positive attitude toward interprofessional learning. This result is consistent with that of a previous study among medical students [38]. Academic self-efficacy has multiple effects on individual development, such as influencing human thinking, goal setting, and learning attitude [39]. Nursing students with higher academic self-efficacy will be more active in daily learning and easily obtain praise and recognition from teachers and classmates. According to the "Rosenthal effect", this external support will increase their confidence in learning, improving their performance in interprofessional learning. In addition, when nursing students with high academic self-efficacy feel a gap in their personal performance and goals, they may develop a strong desire to learn and explore, which is conducive to enhancing their motivation for interprofessional learning. Therefore, academic self-efficacy plays a key role in improving nursing students’ readiness for interprofessional learning.

However, it is noteworthy that senior nursing students in this study had the lowest interprofessional learning readiness scores and the highest academic self-efficacy scores. This relationship has not been found in other studies. Senior nursing students are in clinical internship, and will pay more attention to nursing professional learning, which may result in a lack of time to engage in interprofessional learning.

### Limitations

First, this study only investigated nursing students from one Chinese university, which limits the generalizability of results. A multi-center and large sample survey will be conducted in the future. Second, due to time constraints, this study only conducted a cross-sectional survey for a certain period. This method cannot determine a changing correlation trend between readiness for interprofessional learning and academic self-efficacy. In the future, longitudinal or intervention studies should be conducted to explore the path relationship between these variables. Additionally, this study was limited by COVID-19, and the number of undergraduate nursing students included in the graduating class was relatively small. Future studies must include nursing students who are transitioning to clinical practice. Finally, this study only considered some confounding factors between nursing students' readiness for interprofessional learning and academic self-efficacy. It is suggested to further explore other factors in future studies, such as psychological resilience, academic burnout, and liking degree of nursing profession.

## Conclusions

This study showed that Chinese nursing students were moderately ready for interprofessional learning and academic self-efficacy. There were statistically significant differences in readiness for interprofessional learning scores for nursing students who differed in sex, grade, choice of the nursing profession, and frequency of communication with other health-related major students. There were also statistical differences in the academic self-efficacy scores of nursing students who differed in grade, choice of the nursing profession, and frequency of communication with other health-related major students. Furthermore, we found a positive association between readiness for interprofessional learning and academic self-efficacy.

### Recommendation

Based on the above findings, we suggest that universities have students from multiple majors join the standardized patient-based scenario simulation teaching and project-based learning case teaching programs and assign them different roles and tasks by major, with demographic differences-based interventions. Additionally, nursing educators should begin by improving the academic self-efficacy of nursing students while cultivating their readiness for interprofessional learning.

For example, they can conduct mindfulness training [40] to release students' learning pressure, develop teaching modes such as workshops, and introduce praise education to enhance confidence and concentration in learning. We can promote nursing students’ learning autonomy through a combination of external incentives and internal reinforcement, thereby improving their readiness for interprofessional learning. Then, nursing educators can use the method of pre-course orientation to communicate with students face-to-face before course study. Students are guided to the course from the aspects of course objectives, course nature, content system and learning format, so as to improve their understanding of the learning content, maintain positive beliefs about learning, and prepare for interprofessional learning. Finally, nursing educators can apply a new educational model based on the concept of CDIO (conceive, design, implement and operate) to carefully design the teaching content with academic efficacy as the starting point, so that nursing students can learn and acquire competencies in an active, hands-on, and inter-curricularly connected manner, thus improving their readiness for interprofessional learning.

## Data Availability

The datasets used and/or analysed during the current study available from the corresponding author on reasonable request.

## Abbreviations

TCMUs: Traditional Chinese Medicine Universities
RIPLS: Readiness of Interprofessional Learning Scale
ASES: Academic Self-Efficacy Scale
